# Why so much uncertainty about adjuvant HPV vaccines after local treatment? Can the discrepancy between the positive statistical results and the scientific community doubts be solved?

**DOI:** 10.1186/s13027-024-00572-9

**Published:** 2024-04-04

**Authors:** Paolo Giorgi-Rossi, Maria Lina Tornesello, Franco Maria Buonaguro

**Affiliations:** 1 Epidemiology Unit, Azienda USL– IRCCS di Reggio Emilia, via Amendola 2, 42122 Reggio Emilia, Italy; 2 Molecular Biology and Viral Oncology Unit, Istituto Nazionale Tumori IRCCS Fondazione G. Pascale, via Mariano Semmola, Napoli, Italy

The review article **Can prophylactic HPV vaccination reduce the recurrence of cervical lesions after surgery? Review and prospect** by Han and Zhang, published on October 29, 2023, highlighted the uncertainty about the efficacy of this intervention [1]. In fact, despite several studies showing consistent results in the direction of efficacy, there is still skepticism in the scientific community about the use of the HPV vaccine as an adjuvant therapy, after local treatment, against relapses. Is there a possibility to reduce the uncertainty? To answer this question we should understand why the available evidence is inconclusive. What should be wise public health decision-making? Should the health systems recommend and pay for this intervention or not?

In this debate, we identify **the discrepancies between the statistical uncertainty and biological plausibility** as the main determinant of divergences.

Several studies have shown a protective efficacy of HPV vaccine, with consistent results ranging from 80 to 50% vaccine efficacy [2–4], with few exceptions showing a smaller if any, effect [5, 6]. Most of the studies were under powered [6–8], many of them are not randomized [7, 9] or provide indirect evidence because the vaccine was administered before treatment to already infected women [6, 10, 11], finally, the largest study was based on routinely collected data with too few clinical information to exclude major differences between the compared groups [5]. Furthermore, some studies collected different outcomes at different time points making difficult a sound meta-analysis. Nevertheless, the most recent systematic reviews [2–4] produced consistent estimates of vaccine efficacy for CIN2 + of 50% or more. The statistical uncertainty about the estimates was small enough to exclude the null hypothesis.

The immunological and molecular mechanisms behind the protective role of a preventive vaccine against recurrences (after local treatment) is however still a major scientific problem. The time has passed when doubts were raised about the preventive efficacy and duration of anti-HPV vaccines optimized to induce humoral immunity. However, it is now difficult to explain why such a vaccine could even prevent the lesions from recurring.

Han and Zhang, in addition to the inhibition, by anti-L1 neutralizing antibodies, of the spread of the virus from the removed infected tissue to adjacent cells and/or of new infections due to other cross-reactive HPV genotypes, reconsider the antiviral role of the microenvironment [12, 13]. The surgical intervention and the subsequent anti-inflammatory microenvironment, with a high level of cytokine secretion, could increase the efficacy of post-operative vaccination.

However, although a post-operative vaccination constitutes an effective preventive strategy for women at high risk of new infection due to their promiscuity or a possible state of immunodeficiency, protection against new infection has not an adjuvant relevance and the interval between surgery and vaccination would not constitute an essential requirement.

Thus, in this scientific controversy, the absence of comprehension of the biological mechanism introduces an uncertainty that cannot be overcome by the statistical precision of pooled estimates coming from individually inconclusive studies.

A large randomized trial that can address at the same time efficacy and feasibility, quantify desirable and undesirable effects, as well as costs and implementation requirements will be always claimed. Nevertheless, such a study has not been conducted since the beginning and the ongoing large trials start with a big issue of lack of equipoise. We cannot honestly say that there is genuine uncertainty [14] about the efficacy. We can say that the lack of comprehension about mechanisms prevents us from anticipating the magnitude of the effects, particularly in the long term, but is this sufficient to ethically justify randomization to no vaccine or placebo? Would randomization to an effective intervention or no intervention be justified by the lack of clear recommendations in favor of the intervention? Is it more ethically acceptable in countries where cost effectiveness and sustainability issues prevented for recommending and covering the vaccination? Relativity of ethical issues in clinical research, particularly if justified by lack of resources has been questioned for other interventions [15]. Nevertheless, in preventive interventions the knowledge needs often justify a further gap between the initial proof of efficacy and implementation. In this time elapsing from the evidence of efficacy and public health recommendations, usually we assist to a spread of the intervention following disparate criteria, mostly opportunistic (for example availability of infrastructures or resources) or arbitrary (for example attitude to innovation of local decision makers), but all prone to introduce inequalities. In this landscape would be difficult to say that randomization is a less ethical criterion [16].

Part of the controversy could have been prevented if the initial studies, which started, as usual and almost necessarily, small and underpowered, were conducted to understand both “if” and “how” the intervention works. This point is essential in prevention, where trials are not a direct empirical test comparing all the consequences, in the life span of the woman, of the intervention with the counterfactual of no intervention. Actually, trials testing preventive interventions usually are only a proof of principle of efficacy, and the understanding of underlying mechanisms is necessary to anticipate long-term effects that cannot be reasonably directly observed in time horizon of a trial.

Indeed, new studies and possibly further analyses of the biological samples of previous studies should focus on:

1. differentiating the short term recurrence and the long term risk of CIN2 +;
2. characterizing the HPV DNA integration into host DNA in treated and recurrent lesions;
3. studying the genotype and the genetic differences between HPV detected in the lesion and during post treatment follow up, including recurrences;
4. evaluating prevalence of latent infections in treated women and vaccine efficacy against these infections;
5. defining occurrence of new infections after conization due to the same virus of the treated infection and vaccine efficacy on these new infections.

While waiting for the results of ongoing studies, the health systems and physicians must decide on offering or not the vaccination. Few governmental agencies and scientific societies [4, 17] produced recommendations, ranging from shared decision-making, in the US, to a strong recommendation in favor of public coverage and active offer, in Italy. The inconsistency is the result of different ways to interpret the uncertainty about biological mechanisms and how this uncertainty was integrated in the interpretation of the available evidence. In the most skeptical interpretation, the uncertainty on the mechanism amplified the criticisms about the limits and flows of empirical studies. The panel that recommended the vaccination, instead, considered the consistency between results coming from different studies with different designs (and limits) and between different outcomes measured across the studies as a triangulation sufficient to overcome the lack of biological explanations.

Preventive interventions often present ethical and epistemological dilemmas, highlighting the need for rethinking the role of trials, including the understanding of underlying mechanisms in their secondary objectives, and the way to use direct and indirect evidence in building recommendations.

## Data Availability

Not applicable.
